# Areca nut and its role in oral submucous fibrosis

**DOI:** 10.4317/jced.51318

**Published:** 2014-12-01

**Authors:** Rachana V. Prabhu, Vishnudas Prabhu, Laxmikanth Chatra, Prashant Shenai, Nithin Suvarna, Savita Dandekeri

**Affiliations:** 1 Reader. Department of Oral Medicine and Radiology, Yenepoya Dental College, Yenepoya University, Mangalore, Karnataka, India; 2 Professor. Department of Oral and Maxillofacial Pathology, Yenepoya Detal College and Hospital. Yenepoya University, Mangalore, Karnataka, India; 3 Professor and Head. Department of Oral Medicine and Radiology, Yenepoya Dental College, Yenepoya University, Mangalore, Karnataka, India; 4 Senior Professor. Department of Oral Medicine and Radiology, Yenepoya Dental College, Yenepoya University, Mangalore, Karnataka, India; 5 Professor. Department of Encodontics, Yenepoya Dental College, Yenepoya University, Mangalore, Karnataka, India; 6 Professor. Department of Prosthodontics, Yenepoya Dental College,Yenepoya University,Mangalore, Karnataka, India

## Abstract

Areca nut, commonly called as betel nut or supari, is a fruit of areca catechu palm tree, which is native of South Asia and Pacific Islands. The seed or endosperm is consumed fresh, boiled or after sun drying or curing. Chewing areca nut is thought to have central nervous system stimulating effect and along with this it is known to have salivary stimulating and digestive properties. According to the traditional Ayurvedic medicine, chewing areca nut and betel leaf is a good remedy against halitosis. It is also used for its deworming property. Along with these beneficial effects of areca nut one of its most harmful effects on the human body in general and oral cavity in particular is the development of potentially malignant disorder called Oral Submucous Fibrosis. The present paper discusses in detail the effects of the components of areca nut on pathogenesis of Oral Submucous Fibrosis.

** Key words:**Areca nut, oral submucous fibrosis, potentially malignant disorder, supari.

## Introduction

Areca nut or supariis a fruit of areca catechu palm tree, which is native of South Asia and Pacific Islands. It is commonly called as betel nut as it is often chewed wrapped in betel leaves.While fresh, the husk is green in color and the nut inside is very soft whereas in a ripe fruit the husk becomes yellow or orange and the fruit inside hardens to a wood like consistency (1). The seed or endosperm is consumed fresh, boiled, or after sun drying or curing.Areca nut is chewed by itself or in the form of commercial preparations like supari, mawa, pan masala (2).Areca nut consumed with betel leaf, lime with or without tobacco is called as Paan or betel quid. It may include clove, cardamom, catechu, etc. for extra flavouring (1).

## History

Indian culture and tradition hold areca nut and betel leaves in high esteem. Considered an auspicious ingredient in Hinduism, the areca nut is still used along with betel leaf in religious ceremonies and also while honouring individuals in most of Southern Asia. In the Indian subcontinent the chewing of betel leaf and areca nut dates back to the pre-Vedic period of Harappan Empire. Formerly in India and Sri Lanka it was a custom of the royalty to chew Areca nut and betel leaf. There was also a custom to chew Areca nut and betel leaf among lovers because of its breath-freshening and relaxant properties (3).

## Effects of Areca Nut

Areca nuts are chewed with betel leaf for their effects as a mild central nervous system stimulant (4).The effect is thought due to one of its content known as arecoline that leads to alertness, increased stamina, a sense of well-being and euphoria. It is known to stimulate salivation and thus aiding in digestion. According to traditional Ayurvedic medicine, chewing areca nut is a good remedy for deworming and along with betel leaf it prevents halitosis (5).Along with these beneficial effects of areca nut one of its most harmful effects on the human body in general and oral cavity in particular is the development of apotentially malignant disorder called Oral Sub-mucous Fibrosis (OSF) (6,7).

## Oral Submucous Fibrosis (OSF)

Oral submucous fibrosis (OSF) is a chronic, insidious oral mucosal condition that occurs predominantly among Indians and occasionally in other Asians especially Taiwanese and sporadically in Europeans. It was first described by Pindborg and Sirsat (8). It is regarded as a pre-cancerous condition (7).

The hallmark of the disease is submucosal fibrosis that affects the oral cavity and progressively involves the pharynx and the upper esophagus. It is characterized by juxta-epithelial inflammatory reaction followed by chronic change in the fibro-elasticity of the lamina propria and is associated with epithelial atrophy. This leads to burning sensation in the oral cavity, blanching, and stiffening of oral mucosa and oropharynx, resulting in restricted mouth opening which in turn causes limited food consumption, difficulty in maintaining oral health, and impairs the ability to speak (6,9).

The presence of palpable fibrous bands is a diagnostic criterion for OSF. The fibrous bands occur especially in the buccal mucosa,retromolar areas, and around the rimaoris. When the tongue is affected, it is devoid of papillae and becomes smooth. Its mobility, especially the protrusion, is impaired. The opening of the mouth is restricted. Some investigators adhered to the earlier signs and symptoms such as pain, history of vesicles and ulcers, and blanching of the mucosa for diagnosis of OSMF (10).

Due to this characteristic clinical appearance there are very few conditions that need to be differentiated from it such as scleroderma, anemia and lichen scerosus.

Scleroderma is an autoimmune condition affecting the connective tissue characterized by intense fibrosis of the skin with internal organ involement. Although there is restricted mouth opening, microstomia and xerostomia in these patients, presence of Raynaud’s phenomenon, widening of the periodontal ligament space and osseusresorbtion of the mandible, increased collagen fibers in the deep layers of the dermis and serological tests helps us to differentiate it from OSF (11). Usually pale mucosa seen in anemic conditions may be mistaken for OSF. Presence of habit, palpable fibrous bands, histological findings and hematological examination allow us to differentiate it from anemia (12). Lichen scerosus is a mucocutaneous chronic inflammatory disease of uncertain origin mostly seen in females and predominently affects skin and genital mucosa. Oral involvement is rarely seen. Clinically it appears as an asymptomatic white macule often on the gingiva and labial mucosa. Histological examination is important in differentiating it from OSF that shows hyalinization and connective tissue scerosis with absence of vascular lumen obliteration (13).

Although the pathogenesis of the disease is thought to be multifactorial, chewing of betel quid/areca nut has been recognized as one of the most significant risk factors for OSF (9).

The tremendous rise in the incidence of this condition has been reported. Worldwide estimates in 1996 indicate that 2.5 million people were found to be affected by this disease, whereas in 2002, in the Indian continent alone, the statistics for OSF was about 5 million people (0.5% of the population of India) (14,15).

In recent years marked increase in the occurrence of OSF was observed in many parts of India like Bihar, MP, Gujarat and Maharashtra and the younger generation are suffering more due to incoming of areca nut products in different multicolored attractive pouches (16).

The prevelance of OSF among patients attending dental OPD in Jaipur, Rajasthan, India in 2012 was found to be 3.39% (17).

Therefore OSF is considered as a public health issue in many parts of world including the UK, South Africa, and many Southeast Asian countries (14,15). The reason for the continuous rise in the incidence of OSF could be attributed to increased popularity of commercially available areca nut preparations, i.e., paan masala/gutkha in India and an increased uptake of this habit by young people due to easy access, effective price ranges, and marketing strategies.

The malignant transformation rate of OSF has been reported to be around 7.6% over a 17-year period (7,18).

## Histopathological Features of OSF

The initial pathology of OSF is characterized by juxta-epithelial inflammation, including edema, large fibroblasts and an inflammatory infiltrate, consisting primarily of neutrophils and eosinophils (19).

Later, collagen bundles with early hyalinization are seen and the acute inflammatory infiltrate contains more chronic cell types,such as lymphocytes and plasma cells, occasionally resembling lichenoid mucositis. In more advanced stages, OSF is characterized by formation of thick bands of collagen and hyalinization extending into the submucosal tissues and decreased vascularity. The epithelium lining frequently becomes thin and loses melanin or becomes hyperkeratotic. Occasionally dysplastic changes occur in the epithelium. Inflammation and fibrosis of minor salivary glands canalso be seen. Muscle degeneration occurs in advancedstages of OSF (19).

## Role of Areca Nut in the Pathogenesis of OSF

A number of etiological factors have been discussed to play a role in the pathogenesis of this disease. Factors include areca nut chewing, ingestion of chillies, genetic and immunologic processes, nutritional deficiencies. Various epidemiological studies and histopathological effects on fibroblast and keratinocytes support the chewing of areca nut as one of the most important risk factors for OSF (14).

Areca nut is made up of alkaloid and flavonoid components. Four alkaloids namely arecoline, arecaidine, guvacine, and guvacoline have been identified in areca nut, of which arecoline is the most potent agent and plays a major role in the pathogenesis of OSF by causing an abnormal increase in collagen production. The flavonoid components like tannins and catechinshave been found to have some direct influence on collagen metabolism (14).

**Figure 1:**
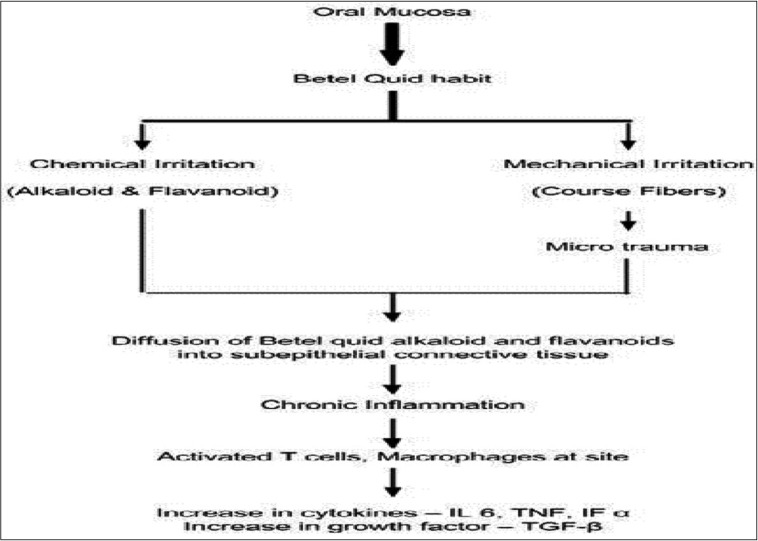
Schematic representation of chronic inflammatory process at the site of placement of the betel quid.

Basic mechanisms involved in the pathogenesis of OSF can be divided into four steps:

1. Occurrence of the chronic inflammation at the site of betel quid placement

2. Increased collagen synthesis

3. Collagen cross-linking

4. Decreased collagen degradation

- Occurrence of the chronic inflammation at the site of betel quid placement:

The areca nut chewing habit varies among individuals. The most common habit is placement of the betel quid in the vestibule for varying duration and frequency. Due to the constant contact between the betel quid and the oral mucosa, the alkaloids and the flavonoidsfrom the quid are absorbed and undergoes metabolism. These constituents along with their metabolites serve as a constant source of irritation to the oral mucosa. Along with this chemical irritation, the mechanical irritation of the oral mucosa also occurs due to the presence of the coarse fibers in the betel quid. The microtrauma resulting from this continues friction of coarse fibers of areca nut facilitates the diffusion of betel quid alkaloids and flavonoids into the subepithelial connective tissue, resulting in juxtaepithelial inflammatory cell infiltration (14). Due to the persistent habit of areca nut chewing, chronic inflammation sets in at the site. Inflammation is characterized by the presence of activated T cells, macrophages, etc. (Fig. 1). Synthesis of various inflammatory mediators like prostaglandins secreted by the oral keratinocytes in response to areca nut extract has been shown (20,21).

Cytokines like interleukin 6 (IL 6), tumor necrosis factor (TNF), interferon α and growth factors like Transforming growth factor – beta (TGF-β) are synthesized at the site of inflammation (22). Initial irritation leads to the further atrophy and ulceration of the mucosa. Persistent inflammation is essential for the occurrence of tissue fibrosis and cancer (23). Hence the induction of oral mucosal inflammation by betel quid ingredients can be considered as a critical step in the pathogenesis of OSF.

-Increased collagen synthesis.

TGF- β is a key regulator of Extra- cellular matrix (ECM) assembly and remodelling. It helps in activation of procollagen genes and elevation of pro-collagen proteinase levels (PCP – Procollagen C-proteinase, BMP 1- bone morphogenetic protein1 PNP – Procollagen N-proteinase).

Procollagen genes are transcribed and translated to form pro-collagen monomeric chains (procollagen precursor). Three of these monomers assemble into a trimer triple helix which is aided by disulphide bridge formation. These trimericprocollagen chains are acted upon by N and C – terminal proteases to cleave the terminal domains and form the fibrils. The newly formed fibrils are then covalently stabilized through cross-linking to form a stable mature structure of collagen (14).

**Figure 2:**
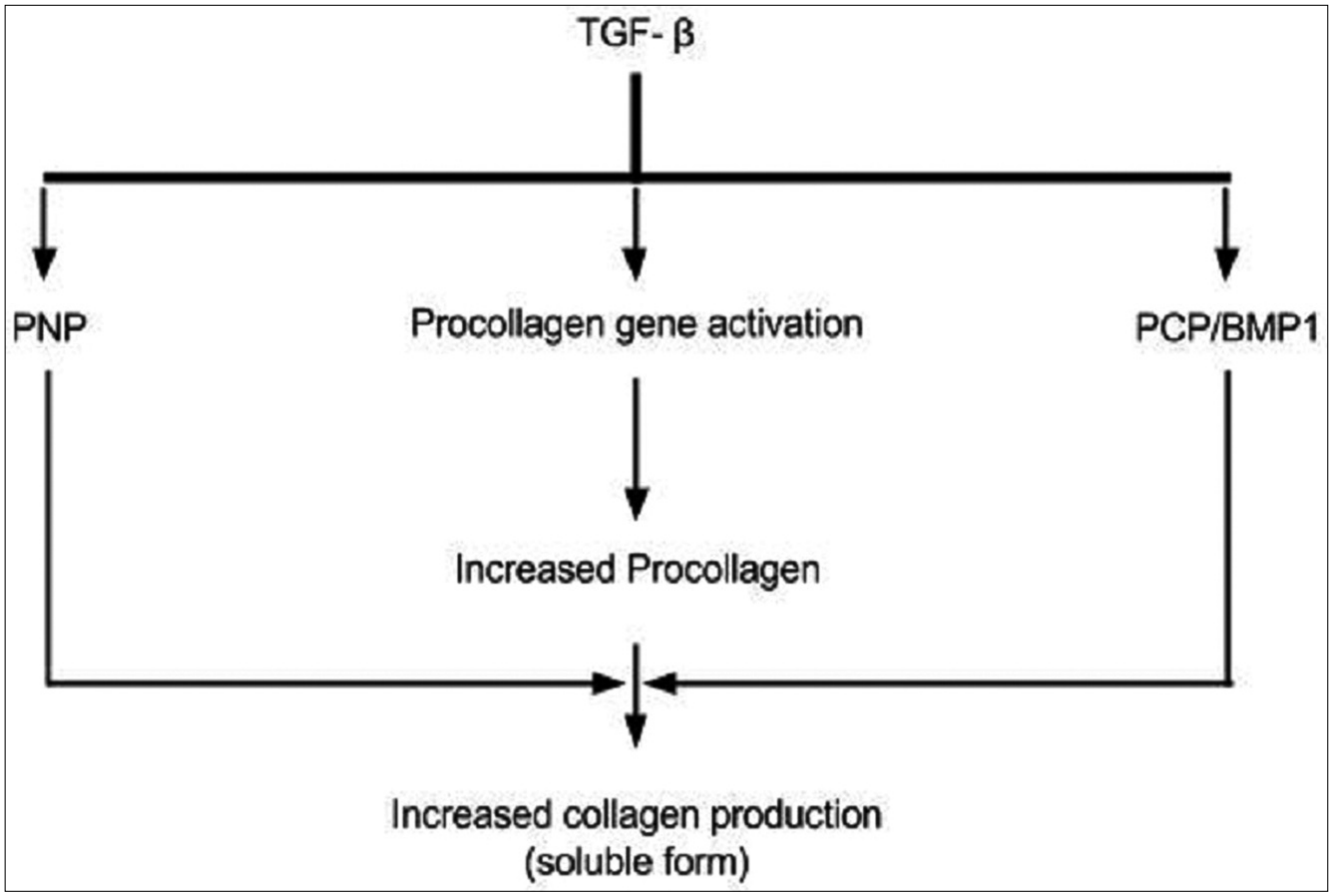
Schematic representation of collagen synthesis regulated by TGF-β.

The genes COL1A2, COL3A1, COL6A1, COL6A3, and COL7A1 have been identified as definite TGF- β targets. The transcriptional activation of types I and type VII collagen gene expression by TGF- β has been demonstrated (23). Due to this it results in increased expression of procollagen genes and thus contributes to the increased collagen levels in OSF (Fig. 2).

Procollagen proteinases (PNP and PCP) helps in processing of procollagen precursors into collagen fibrils, which are soluble. Thus TGF- β modulates both processes i.e. increased procollagen gene expression and processing into fibrils by increased levels and activities of PCP and PNP (14).

The end result is increased collagen synthesis.

- Collagen cross-linkin:

The lysyl oxidase (LOX) is an essential enzyme for final processing of collagen fibers into a stabilized covalently cross-linked mature fibrillar form that is resistant to proteolysis. The activity of LOX leads to the formation of insoluble collagen due to cross-linking. This gives tensile strength and mechanical properties to the fibers and thus makes them resistant to proteolysis.

The LOX is a copper dependent enzyme (24). The conversion of its precursor form i.e. prolysyl oxidase to its active LOX is mediated by BMP1 that occurs in extracellular matrix.(25). Copper is incorporated in LOX during its synthesis (26). Another important co-factor required for the reaction mechanism (cross linking of collagen fibers) of LOX is lysine tyrosylquinone (LTQ) (27). It is a covalently bound carbonyl prosthetic group. It is suggested that copper plays an important role in stabilization of LTQ (28).

High levels of copper have been demonstrated in areca nut. Chewing areca nut for 5 – 30 minutes increases the soluble copper levels in the oral fluids which in turn stimulates fibrogenesis through up-regulation of LOX activity (29,30).

Flavonoids present in the areca nut also play an important role in the process of enhancing the cross-linking of the fibers. It has been demonstrated in in-vitro studies that presence of catechin raises the LOX activity (31,32).

**Figure 3:**
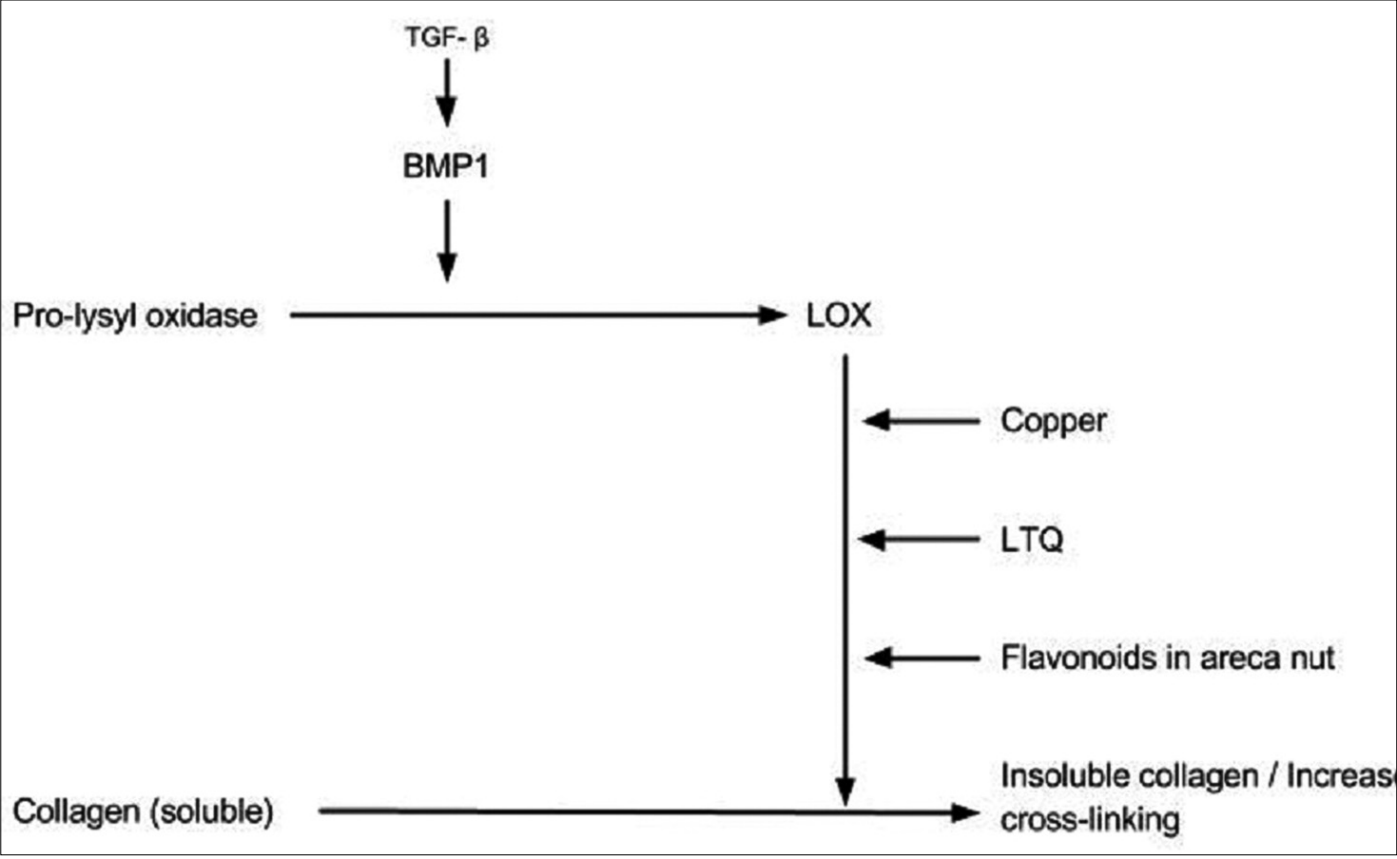
Schematic representation showing the mechanism involved in increased collagen cross-linking.

TGF-β has been considered as an important factor in regulating the expression of LOX. The probable mechanism for this could be elevation of BMP1 by TGF-β which further mediates the bio-synthetic processing of LOX (25) (Fig. 3).

Thus increased LOX activity due to increased BMP and Copper levels and presence of flavonoids in areca nut leads to increased cross-linking of the collagen fibers, that are resistant to proteolysis.

- Decreased Degradation of collagen:

TGF-β decreases the collagen degradation by activation of tissue inhibitor of matrix metalloproteinase gene (TIMPs) and plasminogen activator inhibitor (PAI) gene.

Matrix metalloproteinases (MMPs) comprise of a set of structurally related degrading proteases. Their main function is tissue remodelling by degradation of ECM in health and disease. There are many types of MMPs but few MMPs like MMP 1, MMP8, MMP13 are referred as collagenases. The activities of MMPs are regulated at the level of transcription, activation of the pro-MMPs, and inhibition of endogenous inhibitors under normal physiological conditions (33).

TIMPs are specific inhibitors of MMPs and thus control their local activities in tissues. Studies have demonstrated increased expression of TIMPs in OSF which are responsible for inhibition of collagenase and decrease in collagen degradation (34,35).

**Figure 4:**
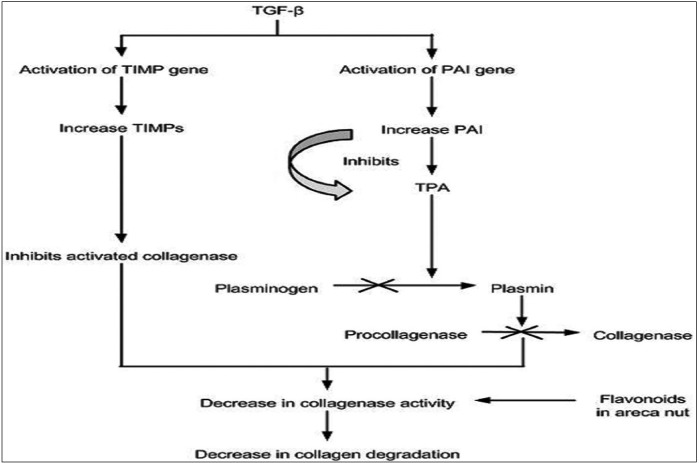
Schematic representation showing mechanisms involved in decreased collagen degradation.

TGF-β activatesTIMP gene thus decreases the collagen degradation by inhibition of the activated collagenases. The flavonoids also have been shown to inhibit the collagenase activity (14) (Fig. 4).

The plasminogen (Plg) activation system is an extra-cellular proteolytic system that plays an important role in tissue remodelling (36). Plasmin is an active serine protease and is generated by proteolytic cleavage of Plg by tissue plasminogen activator (tPA). These activators are regulated by two plasminogen activator inhibitors, PAI1 and PAI2 (37).

Plasmin plays an important role in the activation of pro-MMPs to active form, and thus facilitates the collagen degradation. In OSF, it has been found that there is increase in PAI1 which leads to inhibition of Plg activation process (37) (Fig. 4)

TGF-β has been shown to play a very important role in regulation of PAI1 with its stimulatory effect (38).

Thus there is inhibition of existing collagenase and decreased generation of active collagenase collectively resulting in decrease in collagen degradation.

Apart from activation of TIMPs and PAI genes, another process which is responsible for decreased degradation of collagen is inhibition of collagen phagocytosis. Collagen degradation by fibroblast phagocytosis has animpor-tant role in physiologic collagen turnover. In vitro culture studies have demonstrated that there is reduction in the collagen phagocytic cells in OSF fibroblasts which was thought to be dose dependent with the amount of arecoline/arecaidine (39,40).

## Conclusion

The alkaloid and flavonoid content of the areca nut plays a very important role in the major events that occur in pathogenesis of OSF. They enhance the collagen production, strengthen the cross-linking and reduce their degradation by various discussed molecular pathways. It is a progressive process and hence relieving the patient’s symptoms to improve the life quality forms the basis of its management. Its potential to turn into malignancy is also of concern. It is well said that ‘Prevention is better than cure’ and it holds true in case of the management of this irreversible disease. Thus cessation of the areca nut chewing habit forms the mainstay for the therapy.
